# Disease History and Medication Use as Risk Factors for the Clinical Manifestation of Type 1 Diabetes in Children and Young Adults: An Explorative Case Control Study

**DOI:** 10.1371/journal.pone.0087408

**Published:** 2014-02-03

**Authors:** Soulmaz Fazeli Farsani, Patrick C. Souverein, Marja M. J. van der Vorst, Aukje K. Mantel-Teeuwisse, Catherijne A. J. Knibbe, Anthonius de Boer

**Affiliations:** 1 Division of Pharmacoepidemiology and Clinical Pharmacology, Utrecht Institute for Pharmaceutical Sciences (UIPS), Utrecht University, Utrecht, the Netherlands; 2 Department of Pediatrics, St. Antonius Hospital, Nieuwegein, the Netherlands; 3 Department of Clinical Pharmacy, St. Antonius Hospital, Nieuwegein, the Netherlands; 4 Division of Pharmacology, Leiden Academic Centre for Drug Research, Leiden University, Leiden, the Netherlands; Georgia Regents University, United States of America

## Abstract

**Background:**

There is a highly variable asymptomatic period of beta cell destruction prior to the clinical presentation of type1 diabetes. It is not well known what triggers type 1 diabetes to become a clinically overt disease. This explorative study aimed to identify the association between disease history/medication use and the clinical manifestation of type 1 diabetes.

**Methodology/Principal Findings:**

An explorative case control study was conducted in the Dutch PHARMO Record Linkage System. Cases (*n*  = 1,107) were younger than 25 years and had at least 2 insulin prescriptions between 1999 and 2009. For each case, up to 4 controls (without any prescription for the glucose lowering medications (*n*  = 4,424)) were matched by age and sex. Conditional logistic regression analysis was used to evaluate the association between disease history/medication use in the year prior to the diagnosis of type1 diabetes and clinical manifestation of this disease. Type1 diabetes was significantly associated with a history of mental disorder (odds ratio (OR) 8.0, 95% confidence interval (CI) 1.5–43.7), anemia (OR 5.1, 95% CI 1.1–22.9), and disease of digestive system (OR 2.6, 95% CI 1.2–5.5). The following drug exposures were significantly associated with the clinical manifestation of type 1 diabetes: “systemic hormonal preparations” (OR 1.7, 95% CI 1.1–2.6), medications for “blood and blood forming organs” (OR 1.6, 95% CI 1.1–2.6), “alimentary tract and metabolism” (OR 1.3, 95% CI 1.1–1.6), and “anti-infectives for systemic use” (OR 1.2, 95% CI 1.01–1.4).

**Conclusions:**

Our explorative study demonstrated that in the year prior to the presentation of type1 diabetes in children and young adults, hospitalization for a diverse group of diseases and drug exposures were significantly more prevalent compared with age- and sex-matched diabetes-free controls.

## Introduction

Type 1 diabetes is a chronic disease with a preclinical phase characterized by auto-immunity against pancreatic islet cells ultimately leading to absolute insulin deficiency [1]. Interactions between polygenetic susceptibility and one or more triggering environmental factors are assumed to provide the essential components for this T cell mediated auto-immune disease [2], [3]. While the occurrence of childhood diabetes was stable in the first half of the 20th century, in the second half there was a continuous increase in the prevalence and incidence of type 1 diabetes worldwide, despite genetic stability of the genes related to this disease [4]–[8]. This increase, therefore, is most likely related to changes in the occurrence of risk factors, both host related and environmental [6], [8]–[10].

There have been several studies that focused on the host and environmental risk factors of type 1 diabetes during the prenatal life and early childhood. These studies have shown associations between developing type 1 diabetes and caesarean sections, pre-eclamptic toxemia, maternal age, birth weight, gestational age, infections, short breast feeding, early exposure to dietary cow’s milk proteins and solid food, deficiency of vitamin D, exposure to toxins like N-nitroso compounds, and neonatal eczema [6], [8], [11]–[15]. Although a substantial number of drugs are known to induce hyperglycemia and therefore increasing the risk of diabetes, the influence of medication on the manifestation of type 1 diabetes has not been systematically studied yet [16], [17].

Prior to the clinical presentation of type 1 diabetes there is a highly variable asymptomatic period of beta cell destruction, which can vary between a few months up to several years [18]. It is important to evaluate the triggering factors shortly before the presentation of overt disease thereby providing valuable information on lowering the risk of type 1 diabetes in genetically susceptible young people. As there is limited knowledge on the period before type 1 diabetes becomes a clinically overt disease [6], the aim of this explorative study was to determine whether any association exists between the medication use/disease history in the year preceding the diagnosis of type 1 diabetes and the occurrence of this disease in children and young adults.

## Methods

### Data Source

Data for this study was obtained from the Dutch PHARMO Record Linkage System (RLS) (http://www.pharmo.nl) that comprises community pharmacy dispensing records linked to hospital admissions. Nowadays data from more than 4 million residents of the Netherlands (both rural and urban areas) are collected in the PHARMO RLS which is representative of the Netherlands [19], [20]. Information of these residents is recorded since 1986 and has been used in several pharmacoepidemiologic and outcome studies [20]. The drug dispensing records consist of data on the dispensed drug, the type of prescriber, the dispensing date, the amount dispensed, and the written dose instructions. Date of hospital admission and discharge, together with primary and secondary diagnoses, are recorded in the hospital records. Diagnoses are coded according to the International Classification of Diseases, Ninth Revision, Clinical Modification (ICD-9-CM) (http://icd9cm.chrisendres.com), whereas the drugs are coded according to the Anatomical Therapeutic Chemical codes (ATC codes) (http://www.whocc.no/atc_ddd_index). Hospital diagnoses and drug exposures retrieved from the prescription records in PHARMO RLS have been validated in several studies [21]–[23].

### Study Design and Population

A case control study was conducted between January 1999 and December 2009. All patients younger than 25 years (including children, adolescents and young adults) with at least 2 insulin prescriptions (based on the ATC codes for insulin preparations (A10A) (http://www.whocc.no/atc_ddd_index)) were selected as potential cases. Either the date of first insulin prescription or first hospital admission for type 1 diabetes (based on the ICD-9-CM (http://icd9cm.chrisendres.com)) was selected as the index date. Patients were excluded in case of ever use of oral glucose lowering medications (in order to exclude potential cases of type 2 diabetes), or the use of glucagon prior to insulin (glucagon is usually prescribed in patients with diabetes for the management of hypoglycemia therefore for cases who had prescription of glucagon before insulin, the index date was not clear). For the comparative analysis, up to four controls without any prescription of glucose lowering medications were matched for each case by sex and year of birth at the index date (for 4 cases we only found 3 controls). Both cases and controls were eligible for inclusion in the study only if they had at least 12 months of exposure history before and 12 months follow-up after the index date. All patients with a history of cystic fibrosis (in order to exclude cystic fibrosis-related diabetes) and a history of malignant neoplasms before the index date were excluded from the study.

### Risk Factors

All hospital admissions (based on ICD-9-CM) were identified in both cases and controls in the period 0–12 months prior to the index date. For some disease categories, there was no hospital admission record observed in the database in the period 0–12 months before the index date, therefore they were excluded from the study and analysis was conducted for the 18 remaining categories of the diseases. All codes used for defining these 18 disease categories are presented in table 1. Prescribed medicines were checked at different levels of ATC codes in the period 0–12 months prior to the index date as captured in the PHARMO RLS.

**Table 1:** Codes used to identify diseases in PHARMO RLS.

Disease category	ICD-9-CM codes for hospital admission
All infections	001–137
Overweight and obesity	278.0
Anaemia	280–285
Thalassemia	282.4
Mental disorders	290–319
Epilepsy	345
Eye disorders	360–379
Otitis	381–382
Diseases of the circulatory system	390–459
Acute respiratory infections	460–466
Pneumonia	480–486
Asthma/Chronic Obstructive Pulmonary Disease (COPD)	490–496
Disease of the digestive system	520–579
Appendicitis	540–543
Inflammatory Bowel Disease (IBD) (Crohn's disease and ulcerative colitis)	555–556
Renal/urinary disease	580–599
Disease of the skin and subcutaneous tissue	680–709
Injury and poisoning	800–999

Abbreviations: ICD-9-CM, International Classification of Disease, 9th edition, Clinical Modification.

### Statistical Analysis

Proportions of different categories of hospitalizations and prescribed medicines prior to the index date were calculated for cases and controls. Conditional logistic regression appropriate for a matched study design was used to estimate the odds ratios (ORs) and corresponding 95% confidence intervals (CIs) for the association between the medication use/disease history and the occurrence type 1 diabetes. As this was an explorative study no corrections for type 1 error were applied for multiple comparisons [24]. Furthermore, effect modification by age and sex has been explored. All analyses were conducted using SPSS version 16.0 (SPSS Inc. Chicago, Illinois, USA).

## Results

**Figure 1:**
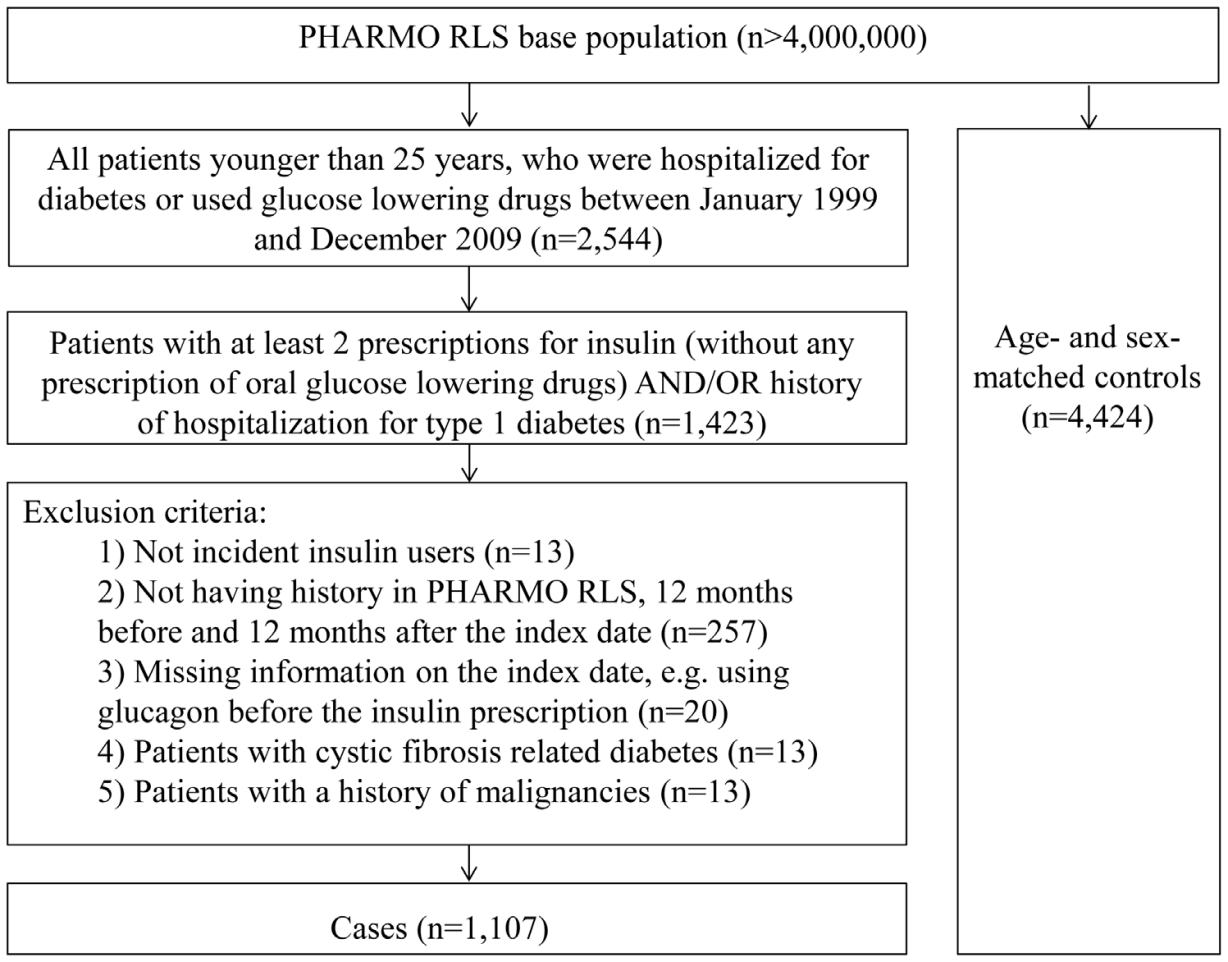
Flowchart of selection of cases and controls.

Of 1,423 patients with at least 2 insulin prescriptions and/or hospital admissions because of type 1 diabetes, 1,107 incident insulin users met the inclusion criteria. At the index date a total of 4,424 controls were matched to these cases by sex and year of birth (Figure 1). The characteristics of the study population are displayed in table 2. The mean age at the index date was 11.8 years [SD 5.8] and 52% of all patients were boys. The majority of the patients with type 1 diabetes (41.5%) were in the 6 to 12 year old category (Table 2).

**Table 2:** Baseline characteristics of patients with type 1 diabetes and diabetes-free controls.

Baseline characteristics		Cases (%) (N = 1,107)	Controls (%) (N = 4,424)
Sex	Male	574 (51.9)	2,297 (51.9)
	Female	533 (48.1)	2,127 (48.1)
Age	0–5 years (infants & preschool children)	175 (15.8)	716 (16.2)
	6–12 years (children)	459 (41.5)	1,817 (41.1)
	13–18 years (adolescents)	304 (27.5)	1,220 (27.6)
	19–24 years (young adults)	169 (15.3)	671 (15.2)
Year of cohort entry	1999–2004	495 (44.7)	1,968 (44.5)
	2005–2009	612 (55.3)	2,456 (55.5)
Mean age [standard deviation]		11.9 [5.8]	11.9 [5.8]

### Association between the History of Other Illnesses and Type 1 Diabetes

Table 3 gives the proportion of patients with hospital admissions in the period 0–12 months prior to the index date for the cases and controls. The total number of hospital admissions in this period was significantly higher in cases versus controls (OR 1.5; 95% CI 1.2–1.9). The strongest associations were observed for mental disorders (OR 8.0, 95% CI 1.5–43.7), anemia (OR 5.1, 95% CI 1.1–22.9), and disease of digestive system (OR 2.6, 95% CI 1.2–5.5) (Table 3).

**Table 3:** Odds ratios comparing history of hospitalization in the cases (patients with type 1 diabetes) with the diabetes-free controls in the period 0–12 months prior to the index date.

Disease category	CasesN = 1,107 N (%)	ControlsN = 4,424 N (%)	OR (95% CI)	p value
All infections	2 (0.2)	6 (0.1)	1.3 (0.3–6.6)	0.725
Overweight and Obesity	1 (0.1)	2 (0.0)	2.0 (0.2–22.1)	0.571
Anaemia	4 (0.4)	3 (0.1)	5.1 (1.1–22.9)	0.033
Thalassemia	1 (0.1)	0	–	–
Mental disorders	4 (0.4)	2 (0.0)	8.0 (1.5–43.7)	0.016
Epilepsy	0	2 (0.0)	–	–
Eye disease	3 (0.3)	6 (0.1)	2.4 (0.6–10.0)	0.231
Otitis	10 (0.9)	48 (1.1)	0.8 (0.4–1.6)	0.594
Acute respiratory infections	0	1 (0.0)	–	–
Pneumonia	2 (0.2)	4 (0.1)	2.0 (0.4–10.9)	0.423
Asthma/COPD	1 (0.1)	2 (0.0)	2.0 (0.2–22.1)	0.571
Disease of the digestivesystem	11 (1.0)	17 (0.4)	2.6 (1.2–5.5)	0.014
Appendicitis	3 (0.3)	3 (0.1)	4.0 (0.8–19.8)	0.09
IBD (Crohn's diseaseand ulcerative colitis)	0	1 (0.0)	–	–
Renal/Urinary disease	3 (0.3)	5 (0.1)	2.4 (0.6–10.0)	0.231
Disease of the skin and subcutaneous tissue	3 (0.3)	9 (0.2)	1.4 (0.4–5.3)	0.605
Injury and poisoning	8 (0.7)	20 (0.5)	1.6 (0.7–3.6)	0.284
Diseases of the circulatorysystem	1 (0.1)	3 (0.1)	1.2 (0.1–11.9)	0.855
Total hospital admissions	95 (8.6)	261 (5.9)	1.5 (1.2–1.9)	0.001

Abbreviations: COPD: Chronic Obstructive Pulmonary Disease; IBD: Inflammatory Bowel Disease; OR: Odds Ratio.

### Association between the Medication Use and Type 1 Diabetes

The following drugs were significantly associated with the clinical manifestation of type 1 diabetes (in order of strength of association): “systemic hormonal preparations, excluding sex hormones and insulins (H)” (OR 1.7, 95% CI 1.1–2.6), “blood and blood forming organs (B)” (OR 1.6, 95% CI 1.1–2.6), “alimentary tract and metabolism (A)” (OR 1.3, 95% CI 1.1–1.6), and “anti-infectives for systemic use (J)” (OR 1.2, 95% CI 1.01–1.4) (Table 4). The most frequently consumed drug categories in patients with type 1 diabetes 0–12 months prior to the index date were “anti-infectives for systemic use (J)” (25.5%), “dermatologicals (D)” (25.2%), “respiratory system drugs (R)” (22.0%), “alimentary tract and metabolism drugs (A)” (13.6%), “sensory organs drugs (S)” (10.0%), “genito urinary system drugs and sex hormones (G)” (9.2%), and “nervous system drugs (N)” (8.8%) (Table 4).

**Table 4:** Odds ratios comparing medication use (anatomical main groups of ATC codes) in the cases (patients with type 1 diabetes) with the diabetes-free controls in the period 0–12 months prior to the index date.

Name of drug category (ATC code)	CasesN = 1,107 N (%)	ControlsN = 4,424 N (%)	OR (95% CI)	p value
Alimentary tract andmetabolism (A)	151 (13.6)	485 (11.0)	1.3 (1.1–1.6)	0.012
Blood and blood formingorgans (B)	26 (2.3)	63 (1.4)	1.6 (1.1–1.6)	0.032
Cardiovascular system (C)	19 (1.7)	72 (1.6)	1.1 (0.7–1.8)	0.737
Dermatologicals (D)	279 (25.4)	1024 (23.1)	1.1 (0.9–1.3)	0.139
Genito urinary systemand sex hormones (G)	102 (9.2)	425 (9.6)	0.9 (0.6–1.3)	0.571
Systemic hormonal preparations, excl.sex hormones and insulins (H)	30 (2.7)	71 (1.6)	1.7 (1.1–2.6)	0.017
Anti–infectives for systemicuse (J)	282 (25.5)	1008 (22.8)	1.2 (1.01–1.4)	0.05
Musculo-skeletal system (M)	61 (5.5)	258 (5.8)	0.9 (0.7–1.2)	0.676
Nervous system (N)	97 (8.8)	349 (7.9)	1.1 (0.9–1.4)	0.368
Antiparasitic products,insecticides and repellents (P)	17 (1.5)	67 (1.5)	1.0 (0.5–1.7)	0.978
Respiratory system (R)	243 (22.0)	988 (22.3)	1.0 (0.8–1.2)	0.803
Sensory organs (S)	111 (10.0)	471 (10.6)	0.9 (0.8–1.2)	0.567

Abbreviations: ATC code: Anatomical Therapeutic Chemical codes; CI, Confidence Intervals; OR: Odds Ratio.

Table 5 provides the proportions of cases and controls with drugs prescribed in the period 0–12 months prior to the index date. These medications are presented in the second (therapeutic main group) and third (therapeutic/pharmacological subgroups) levels of ATC codes. From “alimentary tract and metabolism (A)” category, “antacids (A02A)”, “drugs for peptic ulcer and gastro-esophageal reflux disease (A02B)”, and “drugs for functional gastrointestinal disorders (A03)” were consumed significantly more often in patients with type 1 diabetes than controls. In the cardiovascular category of drugs (C), none of the subcategories were significantly associated with the clinical manifestation of type 1 diabetes. In the “dermatological (D)” group of drugs, “antifungals for dermatological use (D01)” were consumed significantly more often in the cases than controls. “Gynecological anti-infectives and antiseptics (G01A)” were significantly associated with the disease in cases. In the group H, “thyroid preparations (H03A)” were significantly associated with type 1 diabetes. Among anti-infectives for systemic use, proportion of subjects with a prescription for “Antimycotics for systemic use (J02)” was significantly higher in the cases than controls. No significant association between using vaccines and developing type 1 diabetes was found in this study (p = 0.746). “Anxiolytics (N05B)” were used 1.7 times more often in patients with type 1 diabetes. There were no differences between cases and controls in the proportion of “ophthalmological (S01)” and “otological (S02)” prescriptions.

**Table 5:** Odds ratios comparing medication use (therapeutic and pharmacologic subgroups of ATC codes) in the cases (patients with type 1 diabetes) with the diabetes-free controls in the period 0–12 months prior to the index date.

Name of drug category (ATC code)	CasesN = 1,107 N (%)	ControlsN = 4,424 N (%)	OR (95% CI)	p value
Stomatological Preparations (A01A)	37 (3.3)	176 (4.0)	0.8 (0.5–1.2)	0.263
Antacids (A02A)	5 (0.5)	6 (0.1)	3.3 (1.01–10.9)	0.047
Drugs for peptic ulcer andgastro-oesophageal reflux disease (GORD)(A02B)	23 (2.1)	43 (1.0)	2.2 (1.3–3.8)	0.002
Drugs for functionalgastrointestinal disorders (A03)	50 (4.5)	112 (2.5)	1.8 (1.3–2.6)	0.001
Laxatives (A06A)	44 (4.0)	157 (3.5)	1.1 (0.8–1.6)	0.506
Antithrombotic agents (B01A)	2 (0.2)	9 (0.2)	0.9 (0.2–4.0)	0.852
Anti-anaemic preparations (B03)	15 (1.4)	42 (0.9)	1.4 (0.8–2.6)	0.241
Diuretics (C03)	1 (0.1)	0	–	–
Agents for treatment ofhaemorrhoids and analfissures for topical use (C05A)	8 (0.8)	44 (1.0)	0.7 (0.4–1.6)	0.429
Beta blocking agents (C07)	6 (0.5)	16 (0.4)	1.6 (0.6–4.1)	0.346
Calcium channel blockers (C08)	1 (0.1)	2 (0.0)	2.0 (0.2–22.0)	0.571
Agents acting on therenin-angiotensin system (C09)	2 (0.2)	5 (0.1)	1.5 (0.3–7.6)	0.647
Lipid modifying agents (C10A)	1 (0.1)	0	–	–
Antifungals for dermatological use (D01)	80 (7.2)	242 (5.5)	1.3 (1.1–1.8)	0.025
Antipruritics, INCL. antihistamines,anaesthetics, etc. (D04)	8 (0.7)	18 (0.4)	1.8 (0.8–4.1)	0.181
Antipsoriatics (D05)	3 (0.3)	8 (0.2)	1.5 (0.4–5.6)	0.549
Antibiotics and chemotherapeuticsfor dermatological use (D06)	60 (5.4)	258 (5.8)	0.9 (0.7–1.2)	0.533
Corticosteroids, dermatologicalpreparations (D07)	131 (11.8)	449 (10.1)	1.2 (0.9–1.5)	0.087
Anti-acne preparations (D10)	21 (1.9)	102 (2.3)	0.8 (0.5–1.3)	0.435
Gynecological antiinfectivesand antiseptics, EXCL. combinations withcorticosteroids (G01A)	33 (3.0)	46 (1.0)	3.5 (2.1–5.8)	0.000
Sex hormones and modulatorsof the genital system (G03)	91 (8.2)	389 (8.8)	0.8 (0.6–1.2)	0.381
Pituitary and hypothalamic hormones and analogues (H01)	9 (0.8)	36 (0.8)	0.9 (0.5–2.1)	0.970
Corticosteroids for systemicuse, plain (H02A)	11 (1.0)	32 (0.7)	1.4 (0.7–2.8)	0.368
Thyroid preparations (H03A)	10 (0.9)	7 (0.2)	5.5 (2.1–14.5)	0.001
Antibacterials for systemic use (J01)	247 (22.3)	919 (20.8)	1.1 (0.9–1.3)	0.250
Antimycotics for systemic use (J02)	22 (2.0)	26 (0.6)	3.7 (2.0–6.7)	0.000
Antimycobacterials (J04)	0	2 (0.0)	–	–
Antivirals for systemic use (J05)	0 (0.2)	7 (0.2)	1.1 (0.2–5.0)	0.935
Vaccines (J07)	24 (2.2)	93 (2.1)	1.1 (0.7–1.8)	0.746
Anti-inflammatory andantirheumatic products,non-steroids (M01A)	57 (5.1)	249 (5.6)	0.9 (0.7–1.2)	0.527
Anaesthetics, local (N01B)	15 (1.4)	54 (1.2)	1.1 (0.6–2.0)	0.725
Opioids (N02A)	6 (0.5)	11 (0.2)	2.2 (0.8–5.9)	0.124
Other analgesics andantipyretics (N02B)	33 (3.0)	114 (2.6)	1.3 (0.8–1.8)	0.505
Antimigraine preparations (N02C)	2 (0.2)	24 (0.5)	0.3 (0.1–1.4)	0.136
Antipsychotics (N05A)	9 (0.8)	19 (0.4)	1.8 (0.8–4.1)	0.127
Anxiolytics (N05B)	21 (1.9)	49 (1.1)	1.7 (1.1–2.8)	0.044
Hypnotics and sedatives (N05C)	6 (0.5)	20 (0.5)	1.2 (0.5–3.0)	0.712
Antidepressants (N06A)	5 (0.5)	33 (0.7)	0.6 (0.2–1.6)	0.312
Psychostimulants, agentsused for ADHD and nootropics (N06B)	16 (1.4)	72 (1.6)	0.9 (0.5–1.5)	0.669
Antiprotozoals (P01)	9 (0.8)	37 (0.8)	0.9 (0.4–2.0)	0.911
Decongestants and other – nasalpreparations for topical use (R01A)	82 (7.4)	353 (8.0)	0.9 (0.7–1.2)	0.553
Drugs for obstructive airwaydisease (R03)	107 (9.7)	417 (9.4)	1.0 (0.8–1.3)	0.751
Cough and cold preparations (R05)	46 (4.2)	143 (3.2)	1.3 (0.9–1.8)	0.135
Antihistamines for systemic use (R06)	101 (9.1)	381 (8.6)	1.06 (0.8–1.3)	0.602
Ophtalmologicals (S01)	79 (7.1)	314 (7.1)	1.04 (0.8–1.3)	0.904
Otologicals (S02)	38 (3.4)	190 (4.3)	0.8 (0.5–1.1)	0.180

Abbreviations: ATC codes, Anatomical Therapeutic Chemical Classification System; CI, Confidence Intervals; OR, Odds Ratio.

### Interaction with Age and Sex

Because of the low numbers of hospital admissions, it was not possible to check the interaction of age and sex with disease history. Regression analyses indicated that there were no significant interactions with sex and medication use at the first level of ATC codes (anatomical main group), but the interactions between age and drugs in the ATC group A (alimentary tract and metabolism) was significant (p-value: of 0.006). For other medications no significant interaction with age was observed.

## Discussion

This is the first explorative population-based case control study in which disease history (based on the hospital admission data) and drug exposures (based on the prescription data) were evaluated as potential short-term (up to 1 year) triggering factors for type 1 diabetes to become clinically overt in children and young adults. We did not limit our study population to children and adolescents because type 1 diabetes can occur at any age [9]. Our main findings demonstrate that the year prior to the presentation of overt type 1 diabetes in children and young adults, hospitalization for a substantial number of diseases and prescriptions of different drug categories were significantly more prevalent among patients with type 1 diabetes compared with diabetes-free controls. Furthermore, almost all other disease categories for which children were hospitalized such as asthma/COPD, renal/urinary disease, disease of skin and subcutaneous tissues, cardiovascular disorders, etc. (Table 3), although not statistically significant, were positively associated with type 1 diabetes.

Our findings may provide some insight in the potential short-term triggers for the manifestation of type 1 diabetes in children and young adults who are already, and sometimes for years, in a subclinical prodromal phase with diabetes-associated auto-antibodies and beta cell destruction. Such knowledge might be helpful to lower the risk of overt type 1 diabetes in children and young adults already known to be susceptible for this autoimmune disease based on e.g. a positive family history or genetic information. Prevention of certain diseases e.g. some infections or psychological stresses, and optimal treatment may prevent an increased metabolic state and thereby increased insulin requirements. Further research might reveal specific mechanisms and provide further insights into the quantitative risk by which specific diseases and drugs trigger the clinical manifestation of type 1 diabetes. Such studies might also focus on triggering risk factors that occur more than 1 year before the onset of type 1 diabetes. In addition, designing prospective observational studies for finding the environmental risk factors of type 1 diabetes e.g. TEDDY study (The Environmental Determinants of Diabetes in the Young) could benefit from our findings with taking disease history and medication use into account as potential environmental risk factors of type 1 diabetes [25], [26].

Some of the diseases we reported as risk factors for type 1 diabetes in our study have already been published before. Examples are viral infections (at least 13 different viruses were implicated) [13]–[15], gut problems (microbial flora, permeability and mucosal immunity) [15], [27], celiac disease [13], and severe psychological stress [28]. Even though some mechanistic explanations have been proposed, besides that for enterovirus infections, none of those theories are classified as certain [9].

Some of the drugs which we found to be associated with type 1 diabetes are known to influence glucose tolerance and insulin secretion e.g. beta blockers, thiazide diuretics, niacin, corticosteroids, pentamidine, protease inhibitors, atypical antipsychotics [29], calcium channel blockers, cyclosporine, phenytoin, lithium, beta adrenergic agonists, morphine, dapsone, nalidixic acid and rifampicin [30]. For a number of these drugs the mechanism by which this influence occurs has been disclosed. For instance, hypokalemia which induced by thiazides lowers the insulin secretion and corticosteroids enhance the gluconeogenesis, impair glucose uptake by cells and stimulate alpha cells in the pancreas (hyperglucagonemia) [29]. It can be questioned whether these mechanisms are relevant for type 1 diabetes, since type 1 diabetes is an end-stage insulitis which clinically manifests itself when less than 10–20% of the insulin-producing beta cells are still functioning [9]. The way drugs influence glucose tolerance can probably be neglected compared with the critical role of the proportion of functioning beta cells. We hypothesize that a general mechanism by which the diseases and drugs identified in our study may trigger the clinical presentation of type 1 diabetes is that these conditions can change the state of metabolism and thereby increase insulin requirement in the body. Diabetogenic hormones like glucocorticoids and growth hormone may play a role in such a scenario. This increased insulin requirement can induce an earlier presentation of type 1 diabetes and/or an increasing incidence by enlarging the proportion of susceptible children progressing to overt disease. However, we cannot rule out that the increased prevalence of diseases and drug exposures in the year prior to the clinical manifestation of type 1 diabetes can (partly) be explained by a clustering of the diseases (e.g. autoimmune disorders like autoimmune thyroiditis, autoimmune gastritis, pernicious anemia, etc. [31]) or can be caused by the prodromal type 1 diabetes.

The main strength of this study includes its population-based case control design without too many exclusion criteria. Routinely collected detailed data on medication use and hospital admissions reduced the probability of information bias. Also, the use of insulin as a proxy for type 1 diabetes is strong since the treatment of hyperglycemia is the only indication [32], [33]. We assumed that most of the insulin users in our study had type 1 diabetes, because we excluded patients who ever used oral blood glucose lowering medicines, had cystic fibrosis or malignancies in the year prior to the index date, and other types of diabetes needing insulin (e.g. Latent autoimmune diabetes in the adults (LADA) and mitochondrial diabetes, etc.) have low prevalences compared with type 1 diabetes [34], [35]. Therefore misclassification of type of diabetes is probably a minor problem.

Similar to other studies based solely on administrative databases, there are several limitations that must be recognized. It is possible that our results underestimated the prevalence of a number of comorbidities which did not result in hospitalization. While there may be misclassification of drugs and diseases, several studies have validated the exposure of drugs retrieved from the prescription records in the Netherlands and some of the hospital diagnoses have been validated [21]–[23]. Furthermore, information on drugs used in hospitals and over the counter drugs was not available in the current study. Although the PHARMO RLS covers almost 24% of the Dutch population, still the numbers of several drug exposures and hospital admissions were relatively low leading to statistical power problems. Although cases and controls were matched on age and sex, other potential confounding factors like genetic related risk factors, autoimmune antibodies and family history of type 1 diabetes were not available. We cannot rule out that children in the preamble period of the clinical presentation of type 1 diabetes more often visit doctors with an increased chance of identification of diseases and drug prescriptions (ascertainment bias). Finally, there is the problem of multiple comparisons that increases the chance for type 1 errors (false positive findings). However, since this is an explorative study and the general picture is that most diseases and drugs are risk factors for type 1 diabetes we do not think it is necessary to control the family wise error rate (e.g. Bonferroni correction) [24].

In conclusion, it appears that a substantial number of diseases and drugs or the underlying diseases for which these drugs were prescribed were significantly more prevalent among patients who eventually developed type 1 diabetes compared with diabetes-free controls. This knowledge may stimulate further research directed at the prevention of the occurrence and the optimal treatment of these conditions in children and young adults who are susceptible for type 1 diabetes.

## References

[1] Ziegler AG, Schmid S, Huber D, Hummel M, Bonifacio E (2003) Early infant feeding and risk of developing type 1 diabetes-associated autoantibodies. JAMA 290: 1721–1728 10.1001/jama.290.13.1721. 14519706 10.1001/jama.290.13.1721

[2] Atkinson MA, Eisenbarth GS (2001) Type 1 diabetes: New perspectives on disease pathogenesis and treatment. Lancet 358: 221–229 10.1016/S0140–6736(01)05415–0. 11476858 10.1016/S0140-6736(01)05415-0

[3] Herold KC, Hagopian W, Auger JA, Poumian-Ruiz E, Taylor L, et al (2002) Anti-CD3 monoclonal antibody in new-onset type 1 diabetes mellitus. N Engl J Med 346: 1692–1698 10.1056/NEJMoa012864. 12037148 10.1056/NEJMoa012864

[4] Daneman D (2006) Type 1 diabetes. Lancet 367: 847–858 10.1016/S0140–6736(06)68341–4. 16530579 10.1016/S0140-6736(06)68341-4

[5] [Anonymous] (2000) Variation and trends in incidence of childhood diabetes in europe. EURODIAB ACE study group. Lancet 355: 873–876. 10752702

[6] Malcova H, Sumnik Z, Drevinek P, Venhacova J, Lebl J, et al (2006) Absence of breast-feeding is associated with the risk of type 1 diabetes: A case-control study in a population with rapidly increasing incidence. Eur J Pediatr 165: 114–119 10.1007/s00431–005–0008–9. 16211397 10.1007/s00431-005-0008-9

[7] Ma RC, Chan JC (2009) Diabetes: Incidence of childhood type 1 diabetes: A worrying trend. Nat Rev Endocrinol 5: 529–530 10.1038/nrendo.2009.180. 19763122 10.1038/nrendo.2009.180

[8] Svensson J, Carstensen B, Mortensen HB, Borch-Johnsen K (2005) Danish Study Group of Childhood Diabetes (2005) Early childhood risk factors associated with type 1 diabetes–is gender important? Eur J Epidemiol 20: 429–434. 16080591 10.1007/s10654-005-0878-1

[9] Knip M, Veijola R, Virtanen SM, Hyoty H, Vaarala O, et al (2005) Environmental triggers and determinants of type 1 diabetes. Diabetes 54 Suppl 2S125–36. 16306330 10.2337/diabetes.54.suppl_2.s125

[10] Gale EA (2002) The rise of childhood type 1 diabetes in the 20th century. Diabetes 51: 3353–3361. 12453886 10.2337/diabetes.51.12.3353

[11] Haynes A, Bower C, Bulsara MK, Finn J, Jones TW, et al (2007) Perinatal risk factors for childhood type 1 diabetes in western australia–a population-based study (1980–2002). Diabet Med 24: 564–570 10.1111/j.1464–5491.2007.02149.x. 17470192 10.1111/j.1464-5491.2007.02149.x

[12] Akerblom HK, Knip M (1998) Putative environmental factors in type 1 diabetes. Diabetes Metab Rev 14: 31–67. 9605629 10.1002/(sici)1099-0895(199803)14:1<31::aid-dmr201>3.0.co;2-a

[13] Akerblom HK, Vaarala O, Hyoty H, Ilonen J, Knip M (2002) Environmental factors in the etiology of type 1 diabetes. Am J Med Genet 115: 18–29 10.1002/ajmg.10340. 12116173 10.1002/ajmg.10340

[14] Visalli N, Sebastiani L, Adorisio E, Conte A, De Cicco AL, et al (2003) Environmental risk factors for type 1 diabetes in rome and province. Arch Dis Child 88: 695–698. 12876166 10.1136/adc.88.8.695 PMC1719595

[15] Vehik K, Dabelea D (2011) The changing epidemiology of type 1 diabetes: Why is it going through the roof? Diabetes Metab Res Rev 27: 3–13 10.1002/dmrr.1141; 10.1002/dmrr.1141. 21218503 10.1002/dmrr.1141

[16] Blackburn DF, Wilson TW (2006) Antihypertensive medications and blood sugar: Theories and implications. Can J Cardiol 22: 229–233. 16520854 10.1016/s0828-282x(06)70902-3 PMC2528930

[17] Izzedine H, Launay-Vacher V, Deybach C, Bourry E, Barrou B, et al (2005) Drug-induced diabetes mellitus. Expert Opin Drug Saf 4: 1097–1109 10.1517/14740338.4.6.1097. 16255667 10.1517/14740338.4.6.1097

[18] Knip M (2002) Natural course of preclinical type 1 diabetes. Horm Res 57 Suppl 1 6–11: 53305. 10.1159/000053305 11979015

[19] Overbeek JA, Penning-van Beest FJ, Heintjes EM, Gerber RA, Cappelleri JC, et al (2011) Dupuytren's contracture: A retrospective database analysis to determine hospitalizations in the netherlands. BMC Res Notes 4: 402–0500-4-402 10.1186/1756-0500-4-402; 10.1186/1756-0500-4-402. 21992150 10.1186/1756-0500-4-402 PMC3203074

[20] Houweling LM, Bezemer ID, Penning-van Beest FJ, Meijer WM, van Lingen RA, et al.. (2013) First year of life medication use and hospital admission rates: Premature compared with term infants. J Pediatr. 10.1016/j.jpeds.2012.12.014; 10.1016/j.jpeds.2012.12.014. 10.1016/j.jpeds.2012.12.014 23333133

[21] De Bruin ML, van Hemel NM, Leufkens HG, Hoes AW (2005) Hospital discharge diagnoses of ventricular arrhythmias and cardiac arrest were useful for epidemiologic research. J Clin Epidemiol 58: 1325–1329 10.1016/j.jclinepi.2005.04.009. 16291479 10.1016/j.jclinepi.2005.04.009

[22] Herings RM, Stricker BH, de Boer A, Bakker A, Sturmans F, et al (1996) Current use of thiazide diuretics and prevention of femur fractures. J Clin Epidemiol 49: 115–119. 8598504 10.1016/0895-4356(95)00552-8

[23] Lau HS, de Boer A, Beuning KS, Porsius A (1997) Validation of pharmacy records in drug exposure assessment. J Clin Epidemiol 50: 619–625. 9180655 10.1016/s0895-4356(97)00040-1

[24] Rothman KJ (1990) No adjustments are needed for multiple comparisons. Epidemiology 1: 43–46. 2081237

[25] Aronsson CA, Vehik K, Yang J, Uusitalo U, Hay K, et al.. (2013) Use of dietary supplements in pregnant women in relation to sociodemographic factors - a report from the environmental determinants of diabetes in the young (TEDDY) study. Public Health Nutr : 1–13. 10.1017/S1368980013000293. 10.1017/S1368980013000293 PMC4112516 23452986

[26] TEDDY Study Group (2007) The environmental determinants of diabetes in the young (TEDDY) study: Study design. Pediatr Diabetes 8: 286–298 10.1111/j.1399–5448.2007.00269.x. 17850472 10.1111/j.1399-5448.2007.00269.x

[27] Vaarala O, Atkinson MA, Neu J (2008) The "perfect storm" for type 1 diabetes: The complex interplay between intestinal microbiota, gut permeability, and mucosal immunity. Diabetes 57: 2555–2562 10.2337/db08–0331; 10.2337/db08–0331. 18820210 10.2337/db08-0331 PMC2551660

[28] Hagglof B, Blom L, Dahlquist G, Lonnberg G, Sahlin B (1991) The swedish childhood diabetes study: Indications of severe psychological stress as a risk factor for type 1 (insulin-dependent) diabetes mellitus in childhood. Diabetologia 34: 579–583. 1936661 10.1007/BF00400277

[29] Luna B, Feinglos MN (2001) Drug-induced hyperglycemia. JAMA 286: 1945–1948. 11667913 10.1001/jama.286.16.1945

[30] Pandit MK, Burke J, Gustafson AB, Minocha A, Peiris AN (1993) Drug-induced disorders of glucose tolerance. Ann Intern Med 118: 529–539. 8442624 10.7326/0003-4819-118-7-199304010-00008

[31] De Block CE, De Leeuw IH, Van Gaal LF (2008) Autoimmune gastritis in type 1 diabetes: A clinically oriented review. J Clin Endocrinol Metab 93: 363–371 10.1210/jc.2007–2134. 18029461 10.1210/jc.2007-2134

[32] Herings RM, de Boer A, Stricker BH, Bakker A, Sturmans F (1995) A rapid method to estimate the incidence rate and prevalence of insulin-dependent diabetes mellitus in children 0–19 years of age. Pharm World Sci 17: 17–19. 7719274 10.1007/BF01875553

[33] Hsia Y, Neubert AC, Rani F, Viner RM, Hindmarsh PC, et al (2009) An increase in the prevalence of type 1 and 2 diabetes in children and adolescents: Results from prescription data from a UK general practice database. Br J Clin Pharmacol 67: 242–249 10.1111/j.1365–2125.2008.03347.x. 19260863 10.1111/j.1365-2125.2008.03347.x PMC2670382

[34] Guglielmi C, Palermo A, Pozzilli P (2012) Latent autoimmune diabetes in the adults (LADA) in asia: From pathogenesis and epidemiology to therapy. Diabetes Metab Res Rev 28 Suppl 240–46 10.1002/dmrr.2345; 10.1002/dmrr.2345. 10.1002/dmrr.2345 23280865

[35] Martikainen MH, Ronnemaa T, Majamaa K. (2012) Prevalence of mitochondrial diabetes in southwestern finland: A molecular epidemiological study. Acta Diabetol. 10.1007/s00592-012-0393-2. 10.1007/s00592-012-0393-2 22492248

